# A gesture-based design tool: Assessing 2DOF vs. 4DOF steerable instrument control

**DOI:** 10.1371/journal.pone.0199367

**Published:** 2018-07-19

**Authors:** E. A. Arkenbout, J. C. F. de Winter, A. Ali, J. Dankelman, P. Breedveld

**Affiliations:** Department of Biomechanical Engineering, Faculty of Mechanical, Maritime and Materials Engineering, Delft University of Technology, Delft, The Netherlands; Campus Bio Medico University, ITALY

## Abstract

Iterative prototyping is costly and time-consuming. Particularly when designing medical instruments, human factors related design choices significantly impact performance and safety. A tool is presented that allows for the evaluation of steerable instrument controls before the onset of the prototyping stage. The design tool couples gestural input to virtually simulated instrument motions using hand motion tracking. We performed a human-subject evaluation of two manual control strategies that differed in their degrees of freedom (DOF). 2DOF thumb control was compared to 4DOF thumb-index finger control. Results identified regions within the instrument workspace that are difficult to reach and showed participants to favor using the thumb for gross and fine-tuning motions at both control strategies. Index finger ab/adduction was found to be least functional. A strong learning effect was observed at 4DOF control. Based on the results, gesture-based instrument design is a viable design tool.

## Introduction

### Medical instrument design challenges

Many design methods exist [1–4], including general design theory [5], axiomatic design [6, 7], user-centered design [8–11], scenario based design [12–14], participatory design [15], and combinations or variations thereof [16, 17]. Irrespective of the method one employs, prototypes are a necessity in any design process. Only through prototyping are researchers able to set up experiments for testing and evaluation with appropriate user groups. Unfortunately, prototyping is never a one-time event, as design concepts need to be tested, refined, and retested multiple times throughout a development process [18].

Iterative prototyping, particularly in the medical industry, can be a costly and time-consuming process, with no guarantee of eventual market adoption. In the case of surgical instrumentation, clinical evidence towards effectiveness, gathered through clinical trials, ultimately dictates market uptake [19]. Of particular interest are the challenging developments of multi-branched and multi-steerable instruments, such as those developed for Natural Orifice Translumenal Endoscopic Surgery (NOTES) and Single Incision Laparoscopic Surgery (SILS) since 2006 [20]. These instruments are intended to provide the ability to perform surgical procedures or interventions through a natural orifice or via a keyhole incision. Although literature shows that NOTES and SILS can provide advantages for the patient, such as a reduced risk of infections and faster recovery [21, 22], these techniques present challenges to even experienced surgeons with regard to instrument ‘sword-fighting’, triangulation, tissue handling, and bimanual task performance [23].

Multi-branched instruments for minimally invasive applications (including NOTES and SILS) are now being developed [24–27]. Design efforts are primarily focused on increasing the instruments’ maneuvering potential by expanding the incorporated degrees of freedom (DOF) and by allowing for instrument triangulation [28, 29], thereby providing the ability for bimanual task performance. Other developments include human-in-the-loop computer control schemes [28, 30, 31], varying actuation methods [27, 32–34], alternate fabrication methods [35, 36], integrating various functions (e.g., ultrasound) [32, 37], and improving system properties such as stiffness [38], workspace [33], and force-transmission capabilities [33].

Despite ongoing developments, relatively limited adoption is seen for mechanical and robotic multi-branched systems [19]. In an evaluation of the determinants of medical instrument adoption, O’Toole et al. [19] provided the following six factors: (1) clinical need, (2) clinical effectiveness, (3) safety, (4) compatibility, (5) cost, and (6) usability. For robotic instruments these authors observed that all instruments satisfy the first three criteria, but many instruments do not fulfill one or more of the latter three criteria, thus hampering their market uptake. Additionally, issues regarding spatial orientation, ease of use, steep learning curves, operating room limitations, and high costs, are problems that prohibit widespread instrument adoption [19, 39]. We argue that the *usability* factor, which encompasses aspects of system ergonomics, performance, and intuitiveness of use, is currently a limiting factor concerning multi-branched instrumentation development. Indeed, from the literature, it is apparent that most developed multi-branched instruments have not reached clinical practice, which is in part explained due to their control complexities [25, 39].

Control of multi-branched instruments requires either two surgeons to work in concert or a single surgeon to switch between control modes (usually between shaft and branches control). The complex controls may be an indication that insufficient emphasis has been placed on human factors aspects during instrument development, in particular regarding the relationship between (manual) instrument control and the instrument DOF [25]. Human factors research often concerns the assessment and training of residents’ laparoscopic skills proficiency (e.g., [40] [41]). Although assessment and training are essential, a lack of attention to usability during the design process may lead not only to improperly designed instrument controls, but also to human error, and potentially, life-threatening incidents [42]. By adopting human factors principles, medical equipment and its operations may be made safer and more efficient [42].

Considering the existing multi-branched instrument control complexities, incorporating human-centered design principles may focus and expedite the development process such that prototyped instruments better align with natural human control. In this article, we present a design tool that helps to iteratively evaluate steerable instrument controls before the onset of the prototyping stage. The tool allows for a pre-prototyping iterative design optimization concerning instrument controls. Also, we present a proof-of-principle evaluation of two manual control strategies, differing in their number of integrated DOFs.

### Design tool for steerable instrument human factors evaluation

**Fig 1:**
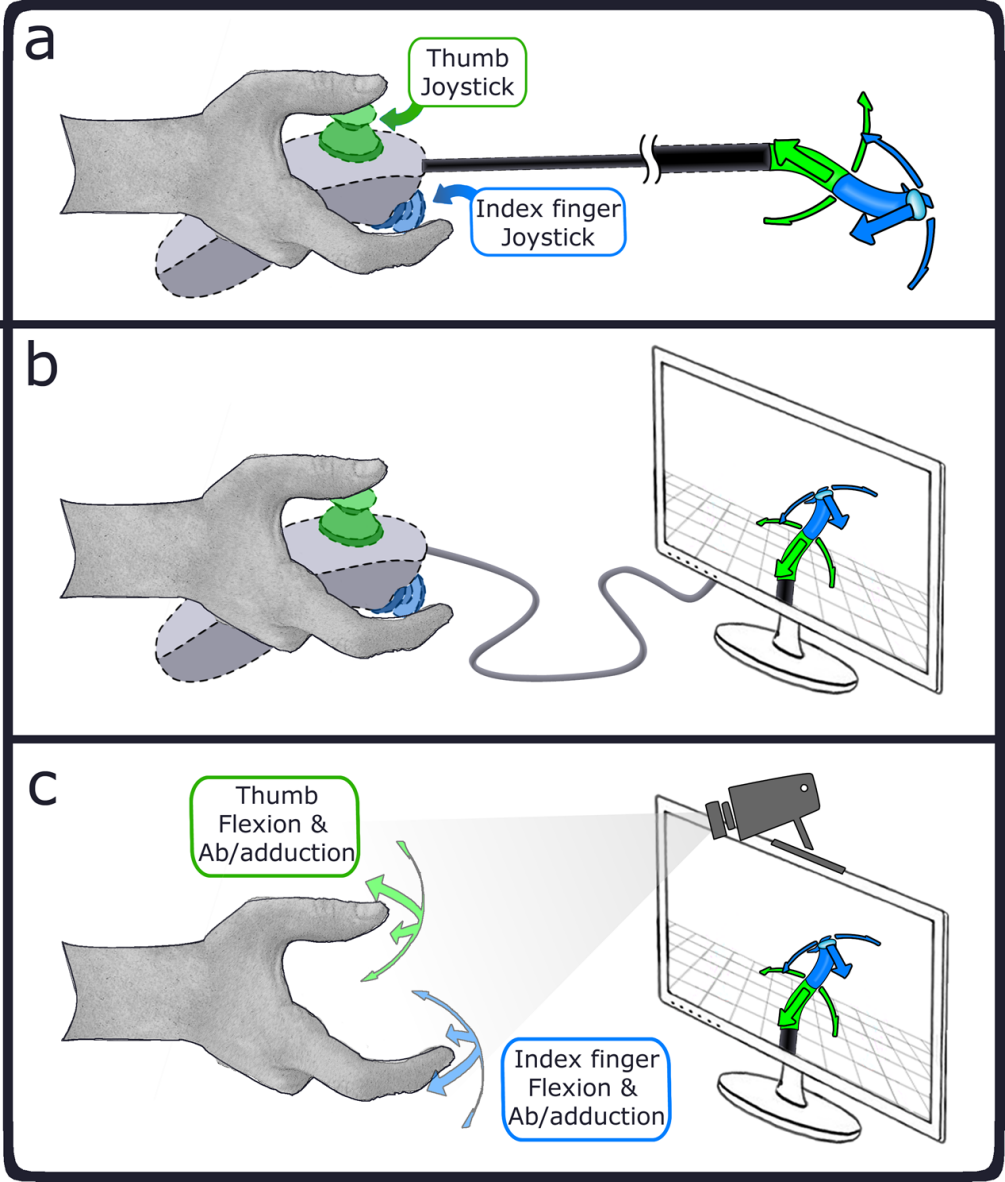
Methods of evaluating control of a theoretical laparoscopic instrument with two stacked 2DOF segments, controlled with the thumb and index fingers. a) evaluation of a physical prototype, with the two segments controlled through joystick; b) physically prototyped controller wired to a computer providing a simulation of an instrument tip; c) control simulation of the theoretical laparoscopic instrument with hand and finger motions measured through camera tracking, with the thumb and index finger motions coupled to two virtual 2DOF segments with a motion mapping strategy congruous to that of the physical instrument.

In this study, we introduce a new design tool for human factors evaluation of steerable instrument control. This section describes the reasoning behind the design tool, using a hypothetical multi-steerable instrument as an example. Fig 1A shows an instrument with at the tip two stacked 2DOF deflecting segments (proximal segment: green; distal segment: blue). Both segments can deflect horizontally and vertically, independently from each other. However, the position of the base of the distal segment is dependent on the tip location of the proximal segment. Each segment connects to a joystick located at the instrument handle. The joystick controlled by the thumb steers the proximal deflecting segment, and the index finger controlled joystick steers the distal segment. The rationale for this instrument lies in its tip maneuvering ability: It can describe non-linear curves (e.g., S-shapes) as well as approach a point in the instrument’s workspace from different angles without having to change the instrument shaft position [43]. The question arises whether an instrument with two 2DOF segments can be effectively controlled manually. To investigate this, one may choose to design and prototype the envisioned instrument and assess this control strategy, but this comes at the cost of considerable time and effort.

Instead of iteratively prototyping during the instrument development cycle, one can virtually simulate the instrument to evaluate design considerations, without the need for extensive prototyping. Instead of providing one with a physical prototype, it is possible to provide instead a physical interface that is congruous with the envisioned instrument and which couples the control inputs to a virtual representation of the instrument, as depicted in Fig 1B. This method was employed in a previous study, where two types of physical controllers were constructed using rapid prototyping and Arduino, and a multi-branched instrument was simulated in virtual reality using the Virtual Robot Experimentation Platform (V-REP) [44]. Due to the virtual nature of the instruments, many design parameters could be tuned during initial pilot tests, including the instrument workspace, magnifications gains, instrument dimensions, bending radius, and triangulation-distance and -angle between instrument branches. The development of the physical controllers, however, remained a time-consuming process. Therefore, the developed test setup was only beneficial for iterative design alterations of the virtually simulated instrument, and not for the physically constructed controllers themselves.

Taking the virtual prototyping design approach one step further, one may consider the option of not only simulating the instrument tip but also simulating the instrument controls without any physical controllers. Recent developments in sensory techniques [45–47] make it possible to measure hand and fingers motion directly. Assuming such measurements are adequately precise and robust to enable human factors analysis, this negates the need for a physical handheld controller altogether. In Fig 1C this method is shown, where the motions the fingers would make when controlling the physical instrument controllers are measured and mapped to the simulated instrument segments. For example, the downward deflection of a (simulated) steerable instrument achieved by pushing down a physical joystick, is instead performed through thumb flexion congruous with the movement the thumb would otherwise make when using the joystick. Although the absence of physical joysticks removes any haptic feedback or tactile cues a person would receive while controlling the physical instrument, the motion mapping strategy is kept as similar as possible. Moreover, any of the input motions may now be changed easily at a moment’s notice.

**Fig 2:**
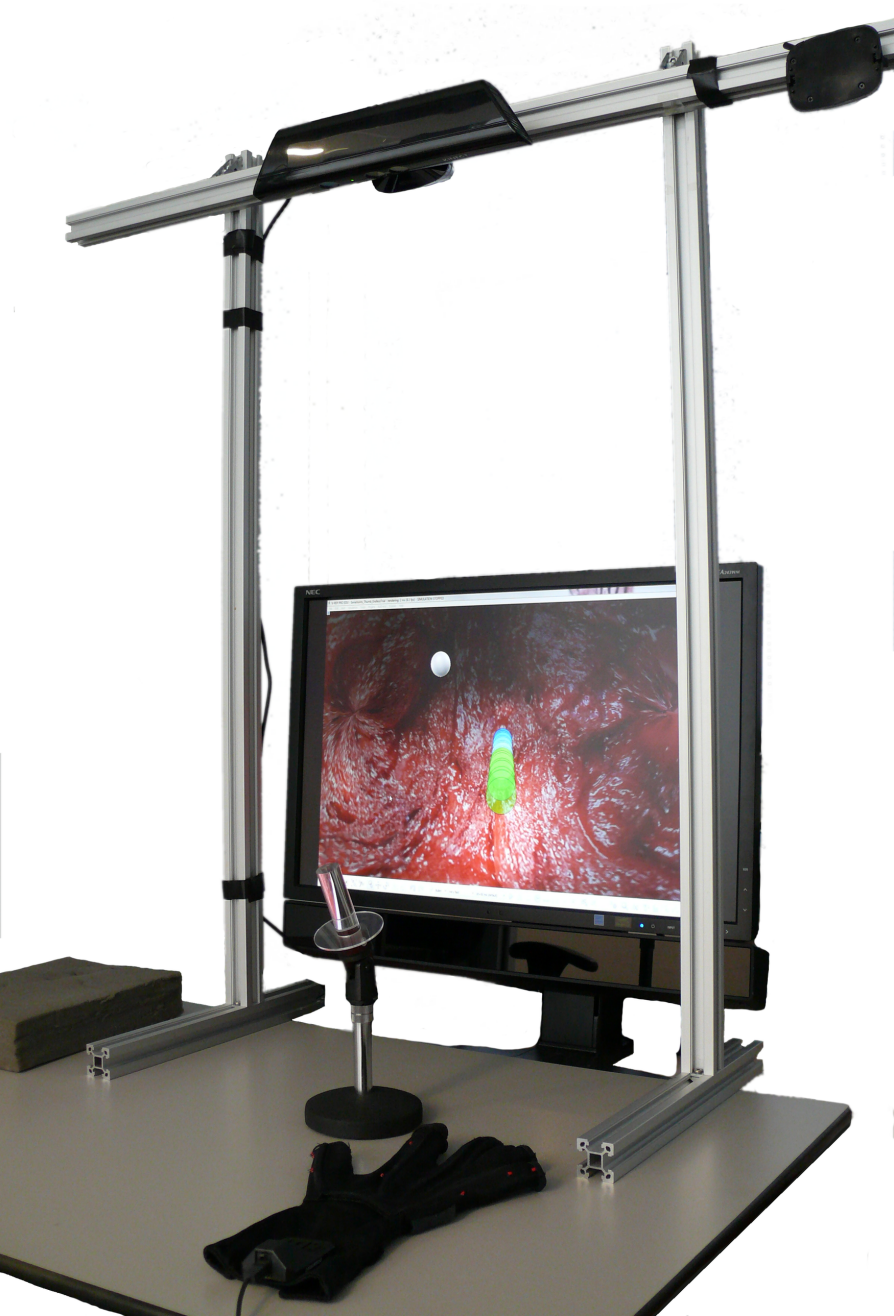
Embodiment of the gesture-based design tool. Photograph of the design tool (with the background removed) incorporating a Kinect camera, Nimble VR camera-based hand tracking software, 5DT Data Glove, and custom written C++ software to fuse the sensory information through a Kalman filter to obtain hand postural information.

A setup that enables tracking of hand and finger motions, by fusing together Nimble VR software camera-based hand tracking [48] and 5DT Data Glove [49] measurements, was developed in a previous study [50]. This setup is implemented in the current research in tandem with a simulation interface to encompass our proposed design tool as shown in Fig 2. The system was determined to have an overall tracking precision of 2.2 deg and 0.9 deg for the metacarpophalangeal (MCP) and proximal interphalangeal (PIP) joints of the fingers, respectively [50]. A 5DT Data Glove, providing five basic full finger flexion sensors (one for each finger), was used to improve data robustness, account for visual (self)occlusions, increase resolution and reduce measurement latency during general finger flexions. A decision was made *not* to use a 5DT Data Glove 14 Ultra, which has 14 sensors to measure MCP and PIP joints flexions and ab/adduction of the fingers [51], as this large number of sensors was deemed obtrusive. The disadvantage of not using the 14 Ultra glove, however, is that in the current setup the measurement of fingers ab/adduction relies on solely the Nimble VR camera-based tracking, whereas finger flexions are measured using both systems combined. Accordingly, a tracking latency of 500 ms is present for ab/adduction of the fingers, and 75 ms for measurements of finger flexions.

The design tool was connected to V-REP, an open source framework that allows 3D CAD models to be imported and assembled, and joints to be defined within specified ranges of motion [52]. Measured hand and finger motions can be mapped to any of the virtually defined segments or joints, so that numerous control coupling strategies can be defined and tested. Human factors assessments can be performed similar to those performed in existing physical benchtop simulators [53], but with improved data gathering potential.

A proof-of-principle evaluation was conducted of the suggested 4DOF instrument, shown in Fig 1. Control of the two 2DOF segments, using the thumb and index fingers, was compared to a similar instrument with only a single 2DOF deflecting segment at its distal end, only controlled by the thumb.

## Materials and methods

### Control strategies

**Fig 3:**
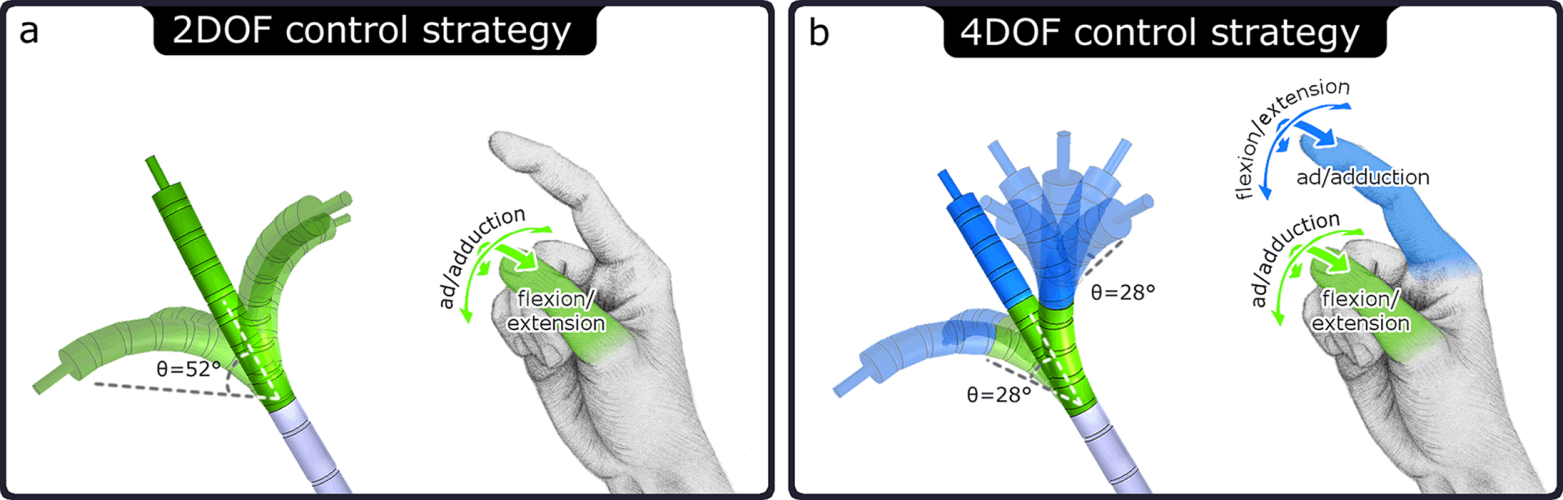
Schematic representation of the evaluated control strategies with the gesture-based design tool. a) 2DOF control strategy, with thumb ab/adduction and flexion/extension coupled to 2DOF tip deflection. b) 4DOF control strategy, with thumb control of proximal tip segment the same as motion coupling in (a), and index finger control of distal segment, with ab/adduction and flexion/extension coupling. Finger motion input and instrument motion output are in the same planes of motion.

Thumb and index finger flexion/extension and ab/adduction movements were mapped to virtual instrument joint rotations in V-REP. Two control modes were compared:

1. 2DOF control strategy. Thumb flexion is coupled to tip bending in the vertical plane and thumb ab/adduction is coupled to bending in the horizontal plane (Fig 3A). When the hand is held in the posture as indicated in Fig 3, and aligned in the facing direction of the instrument, the thumb and instrument movements lie in same planes of motion (e.g., a thumb movement to the left equals instrument bending to the left).
2. 4DOF control strategy. The instrument has two stacked 2DOF deflecting segments as shown in Fig 1C. The thumb controls the proximal segment identical to the 2DOF control strategy. Index finger movements are coupled to the distal segment, where finger flexion controls the bending in the horizontal plane and finger ab-/adduction the bending in the vertical plane (Fig 3B). Similar to the 2DOF control strategy, the finger movements correspond with the instrument deflections in the same directions in the same planes of motion.

The input ranges of motion of the thumb were 40 deg ab/adduction and 50 deg flexion/extension, and for the index finger were 20 deg ab/adduction and 40 deg flexion/extension. Finger flexions were required to be predominantly performed with the PIP joints in a fashion similar to the way one would naturally handle the physical joysticks as displayed in Fig 1A, and participants in the user trials were instructed to do so. MCP joint movements were, however, also measured and functional, because the full finger flexions were used as input for coupling to the virtual instrument joints. Finger joint angles outside their specified ranges of motion were disregarded, rounded to the nearest workspace boundary value, and the zero-positions of the fingers were equal to half their ranges (e.g., index finger zero position = 10 deg abduction & 20 deg flexion). The bending range of the proximal and distal segments at both the 2DOF and 4DOF control strategies was 56 deg. On account of the curve both segments make when bending (rather than being a rigid beam), the overall instrument bending range was 104 deg (instead of 112 deg), see Fig 3. The instrument workspaces were identical for both control strategies. The magnification gains from finger bending to virtual segment deflections were for the 2DOF control strategy 2.6 (= 104/40) and 2.08 (= 104/50) for thumb ab/adduction and flexion, respectively. The ab/adduction and flexion gains at the 4DOF control strategy for the thumb were 1.4 (= 56/40) and 1.12 (= 56/50), and for the index finger 2.8 (= 56/20) and 1.4 (= 56/40), respectively. These gains were selected based on pilot trials participants feedback. Note that for both control strategies, the instrument consists of a proximal and distal segment. In the case of the 2DOF strategy, the thumb movements are identically mapped to both segments to make the comparison of segment movements valid between control strategies. The coupling of thumb movements to the proximal segment is thus identical between control strategies.

### User trials

Assessment of the two control strategies was performed through trials in which participants controlled the virtual instrument to perform multiple positioning tasks. Fourteen persons, of which 9 men and 5 women, aged between 22 and 33 years (mean = 27.4, SD = 2.9) participated in the experiment. Half the participants started with the 2DOF controller (4 men, 3 women), the other half with the 4DOF controller (5 men, 2 women). A single positioning task entailed moving the instrument tip and briefly (for 100 ms) touch a target-sphere that was positioned in the instrument’s workspace. After completing a task, the target was relocated to a new position, indicating the start of the next positioning task. Participants were orally instructed to perform each task as fast as possible and provided with a time score after each measurement round to motivate them to beat their own scores. The simulation entailed the use of a 5 mm diameter instrument, approaching target spheres having a diameter of 5 mm. The endpoint of the instrument with which the targets needed to be touched had a diameter of 1 mm, such that a relative minimum targeting accuracy of 3 mm (= (5+1)/2) was required to complete a task. A maximum duration per task was set to 20 s.

User trials started with a short familiarization and calibration phase, followed by ten rounds, alternating between both control strategies. The familiarization phase consisted of 10 target tasks, divided throughout the workspace, but with no time constraint. Each round consisted of 61 target tasks. No rest break was offered between trials unless participants indicated they required such. However, a workload assessment form that was required to be completed after each round provided a minimum of 1 minute downtime. The sequence of the 61 targets was pre-generated, randomly distributed throughout the workspace, and kept identical between rounds and control strategies. The travel distances from one target to the next varied between 10, 20, 30 and 40 mm, each distance occurring 15 times (i.e., 4 travel distances * 15 targets per distance). The 1st of the 61 targets of every round was negated because it did not have a prior target, and therefore was not associated with a specific travel distance to get there. For each control strategy, 5 rounds were performed, totaling to 610 target tasks (2 control strategies * 5 rounds * 61 tasks per round).

The positioning tasks were identical between control strategies. For the 4DOF control strategy, the position of the targets within the instrument’s workspace dictated whether all 4DOF were required to reach the target, or that the control of a single 2DOF segment would suffice. Targets closer to the center of the workspace could be reached using a single segment (i.e., either the thumb or index finger), whereas targets along the outer edges of the workspace could only be reached by bending both segments, thus requiring the use of both the thumb and index finger to reach the target successfully.

### Ethics statement

The research adhered to the tenets of the Declaration of Helsinki. This study was approved by the Human Research Ethics Committee of the Delft University of Technology. All individuals gave their written informed consent.

### Calibration and error detection

Calibration was performed for both the Data Glove and the Nimble VR system. First, the Data Glove was calibrated using the auto-calibrate function provided in the 5DT Data Glove SDK, automatically scaling sensor readings to the maximum ranges of motion of the participants’ fingers. Auto-calibrate was subsequently turned off for the remainder of the trial.

To calibrate the Nimble VR system, participants were asked to spread both hands in view of the Kinect camera such that the software would calibrate to the proper hand scale (Hs) [54]. Considering that the degree of measured overall finger flexion is underestimated for small hands by approximately 22% in the current setup [50], finger flexion measurements of participants having Hs ≤ 0.82 were scaled up 22%. This was necessary to bring these participants’ sensor readings up to par with those of participants having medium or large sized hands. Note that, as women generally have smaller hands than men, this scaling method was purposely chosen, rather than excluding those women from the trials.

Participants were asked to grasp a support handle (Fig 2) with the middle-, ring and little finger, to prevent fatigue and keep the hand in place (otherwise participants would be required to keep the hand up in midair throughout the trial). The support handle was made from infrared-translucent Plexiglas, such that it would not influence the Nimble VR hand posture estimates. Erroneous measurements intermittently occurred during the trials, either due to participants releasing the support handle or due to sensor noise causing the Nimble VR software to obtain a wrong hand posture estimate. To automatically detect these instances, the hand position and orientation associated with holding the support handle were recorded as a zero-reference at the start of the trial. By comparing live measurements during the positioning tasks against the pre-recorded zero-reference data, erroneous measurements associated with gross deviations in hand position or orientation could be detected. The angular thresholds for pitch, yaw and roll were 40, 30, and 45 deg, and the positional threshold was 50 mm in each direction. Tasks during which hand deviation errors or significant time delays (i.e., >100 ms between measurement updates from the hand tracking measurement system) occurred were removed from the data analysis. The task error rate was 3.5%, which corresponds to approximately 2 out of 61 tasks per round that were discarded on account of errors disrupting the normal task performance.

### Data analysis

Instrument joint angles, instrument tip positions, target positions, and time were recorded at 30 Hz. Two-way repeated measures ANOVA (with independent variables: controller versus round numbers and controller versus travel distance to target) and paired sample t-tests were performed for comparisons between control strategies, rounds, and target distances. A significance level of 0.05 was deemed statistically significant. Data were calculated per person, and later again averaged over all participants. Reported standard deviations (SD) are the deviations of the means across participants. After each round, participants were asked to complete a NASA Task Load Index (TLX) workload assessment [55], and at the end of the trial a System Usability Scale (SUS) assessment [56]. The TLX scores are expressed as percentages, and range from Very Low (0%) to Very High (100%) for the mental demand, physical demand, temporal demand, effort, and frustration items, and from Perfect (0%) to Failure (100%) for the performance item.

Based on pilot trials, it was deemed unlikely for participants to reach the end of their (presumed) asymptotic performance level within 5 rounds (equaling roughly 90 minutes). Lengthening the trial, however, would probably cause fatigue. To indicate a level of performance one would be able to achieve with extensive training, the first author of this publication, who had become proficient in the task, performed 25 rounds with each control strategy (totaling 3050 target tasks) to provide a ‘trained’ performance reference value. S1 Video showcases the use of the design tool for both control strategies side by side throughout the performance of a 100 consecutive tasks performed by the first author of this publication.

## Results

Fourteen persons, of which 9 men and 5 women, aged between 22 and 33 years (mean = 27.4, SD = 2.9) participated in the experiment. Hs as detected by the Nimble VR software, ranged from 0.79 to 0.88 (mean = 0.84, *SD* = 0.03). Data was scaled up by 22% for three female participants having Hs < 0.82, and belonging to different controller groups (i.e., one started the test with the 2DOF controller, the other with the 4DOF controller). All participants performed the full user trails except one participant who was unable to complete the fifth round due to time shortage. Also, the first round using the 4DOF control strategy of another participant failed to record properly.

### Learning effects

**Fig 4:**
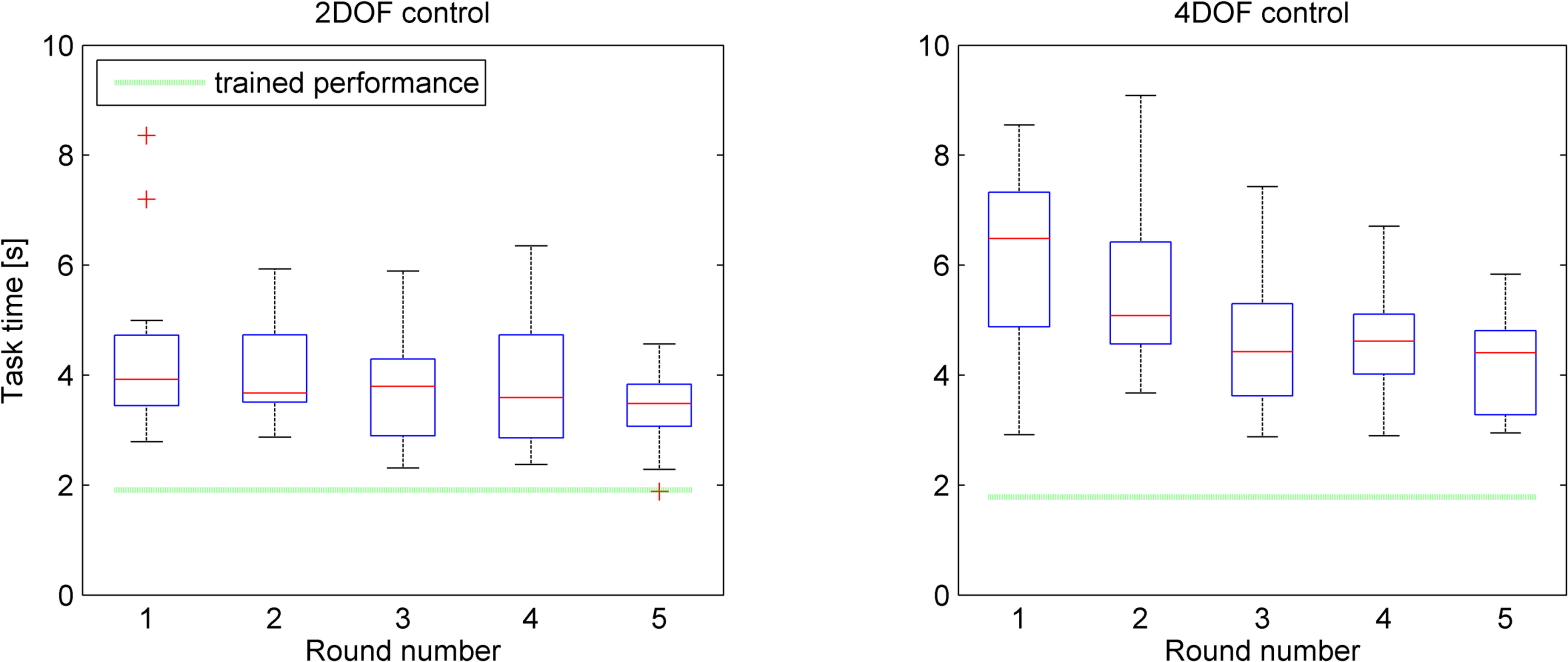
Boxplot of task times with respect to round numbers for both control strategies (n = 14). The dotted line shows for comparison the performance as achieved by the first author after extensive training.

The mean task times for each round and control strategy are provided in Fig 4. Taken across all rounds, the mean task times were 3.89 s (*SD* = 0.94 s) and 5.06 s (*SD* = 1.19 s) for the 2DOF and 4DOF control strategies, respectively.

A learning effect was observed, where the tasks were completed more quickly at later rounds. For the 4DOF control strategy, there was a statistically significant effect of round number (2DOF: *F*(4,8) = 2.61, *p* = .115; 4DOF: *F*(4,8) = 9.63, *p* = .004). A significant interaction was observed between control strategy and round number on mean task performance time, indicating that the 4DOF condition exhibited stronger learning than the 2DOF condition (*F*(4,44) = 3.07, *p* = .026). The task times at the final round for the 2DOF and 4DOF control strategies were 3.34 s (*SD* = 0.72 s) and 4.19 s (*SD* = 0.97 s), respectively, this difference being statistically significant (*t*(12) = -3.24, p = .007).

For comparison, the performance times reached by the first author of this research through extensive training were 1.91 s and 1.78 s for the 2DOF and 4DOF strategy, respectively.

### Influence of travel distance to target

**Fig 5:**
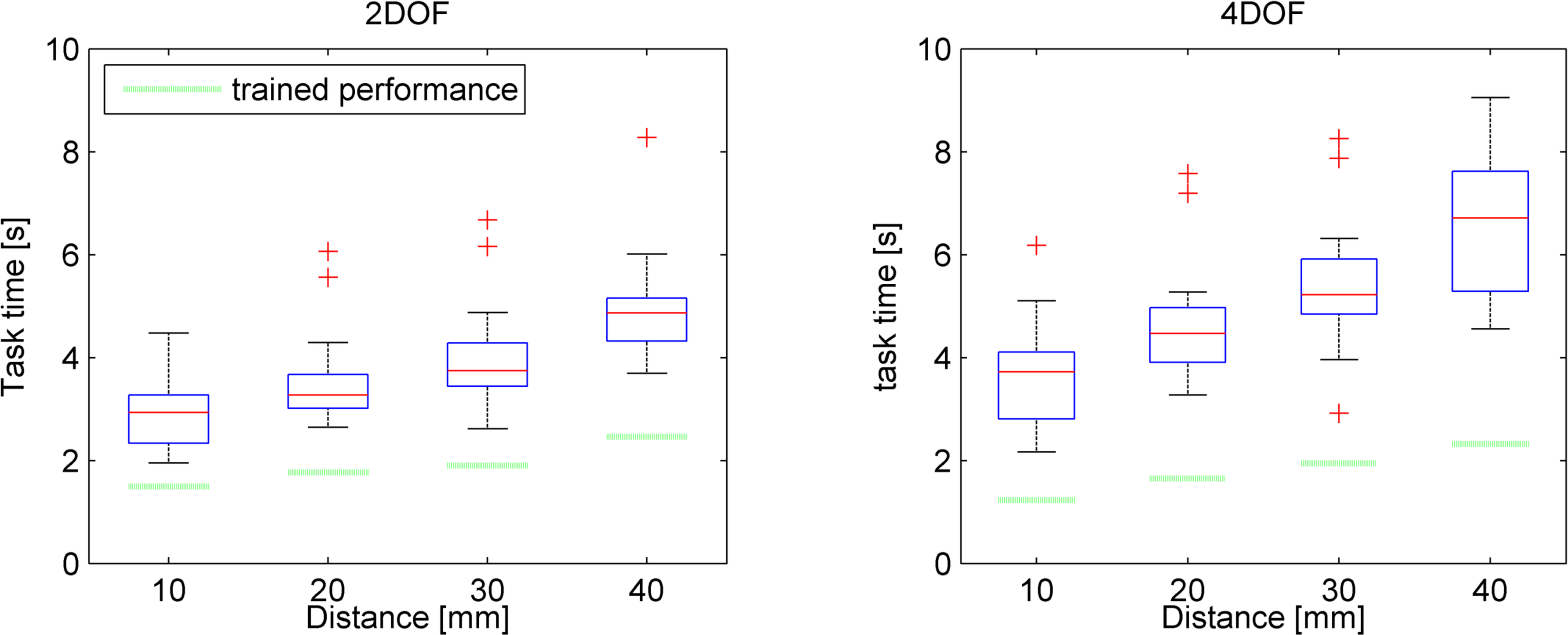
Boxplot of task times as a function of travel distance to targets from previous task’s target and control strategies. The horizontal dashed line represents the performance of the first author after extensive training.

The distances from a previously reached target to the next target varied (10, 20, 30 or 40 mm). Fig 5 shows the task times as a function of their respective target distances for both control strategies.

Two-way repeated measures ANOVAs showed a significant within-subject effect on task time between control strategies (*F*(1,13) = 64.79, *p* < .001) as well between all target distances (*F*(3,39) = 103.64, *p* < .001), with larger distances corresponding to longer task times.

The performance of the first author, also shown in Fig 5, was on average 51.1% lower than the participants’ performance for the 2DOF control strategy, and 62.0% lower for the 4DOF control strategy.

### Influence of target location within workspace

**Fig 6:**
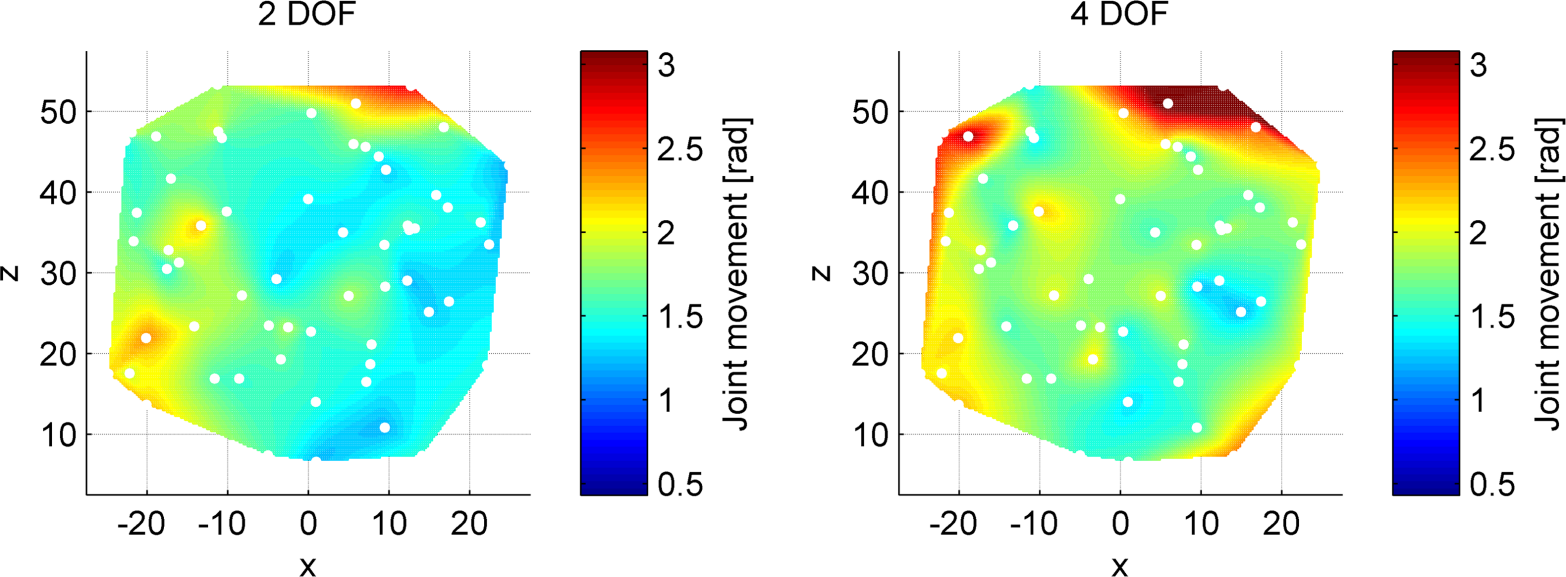
Mean sum of joint movements of proximal and distal instruments segments combined, normalized with respect to the distance to target and learning curve. The two heat maps have the same data scale. The white dots represent the locations of the various targets throughout the workspace.

Heat maps of the mean of joint movements to reach the targets within the 3D instrument workspace are provided in Fig 6, with the data normalized with respect to the target distance and learning curve. Specifically, for each participant, data were normalized by taking the sum of joint movements towards each target in each round, dividing these by the round means of joint movement for a target of that respective travel distance, and multiplying the resulting dimensionless values by the overall trial mean of the sum of joint movements (i.e., the grand mean taken over all rounds). These heat maps represent the degree of movement and corrections made to reach a target. The displayed data in the heat maps is the mean of the normalized data over all participants and rounds.

The heat maps show areas of varying difficulty to reach. For the 2DOF control, the top right and to a lesser extent the bottom left area’s show increased instrument joint movements to reach them. For the 4DOF control, the top right and left areas appear more difficult to reach, and to a lesser extent, the edges of the workspace.

**Fig 7:**
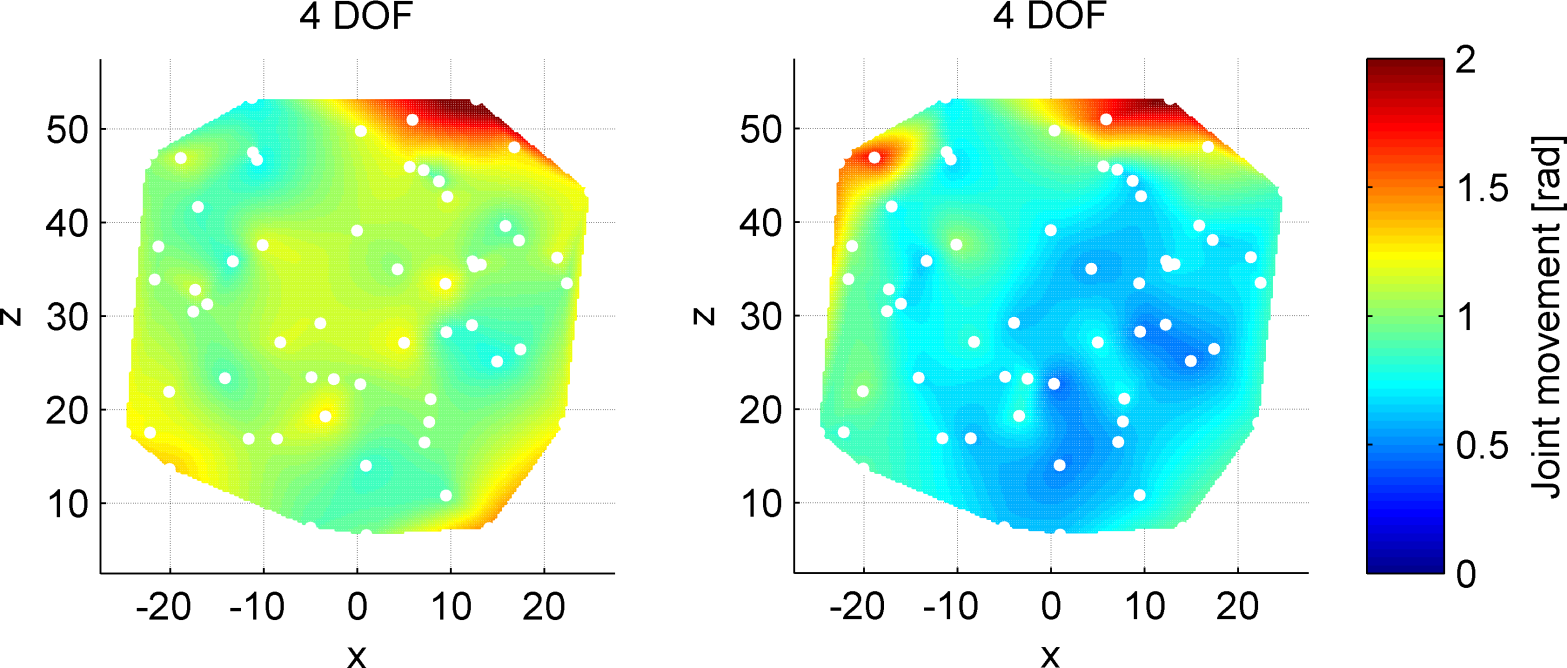
Mean sum of joint movements for the 4DOF control strategy, divided into separately controlled segments. Left: proximal (i.e., thumb-controlled) segment. Right: distal (i.e., index-finger controlled) segment. The two heat maps have the same data scale. The white dots represent the locations of the targets throughout the workspace.

Separating the joint movements at the 4DOF control to their respective segments gives the heat maps as shown in Fig 7. The index finger controlled segment (right plot) shows fewer movements as compared to the thumb-controlled segment (left plot). Additionally, the thumb-controlled proximal segment shows more joint movement at 4DOF control, as compared to the same segment controlled at 2DOF control. The same regions of difficulty for the thumb, however, can be observed at both control strategies. These results indicate the thumb to be predominantly used during target acquisitions at the 4DOF control strategy, and the index finger to only be used when necessary. Note that targets located at the outer edges of the workspace require both segments to be used, thus necessitating index finger use at the 4DOF control strategy. The targets that require extensive index finger abduction are associated with increased distal segment joint movements, and for the remainder, the distal segment is kept passive.

**Fig 8:**
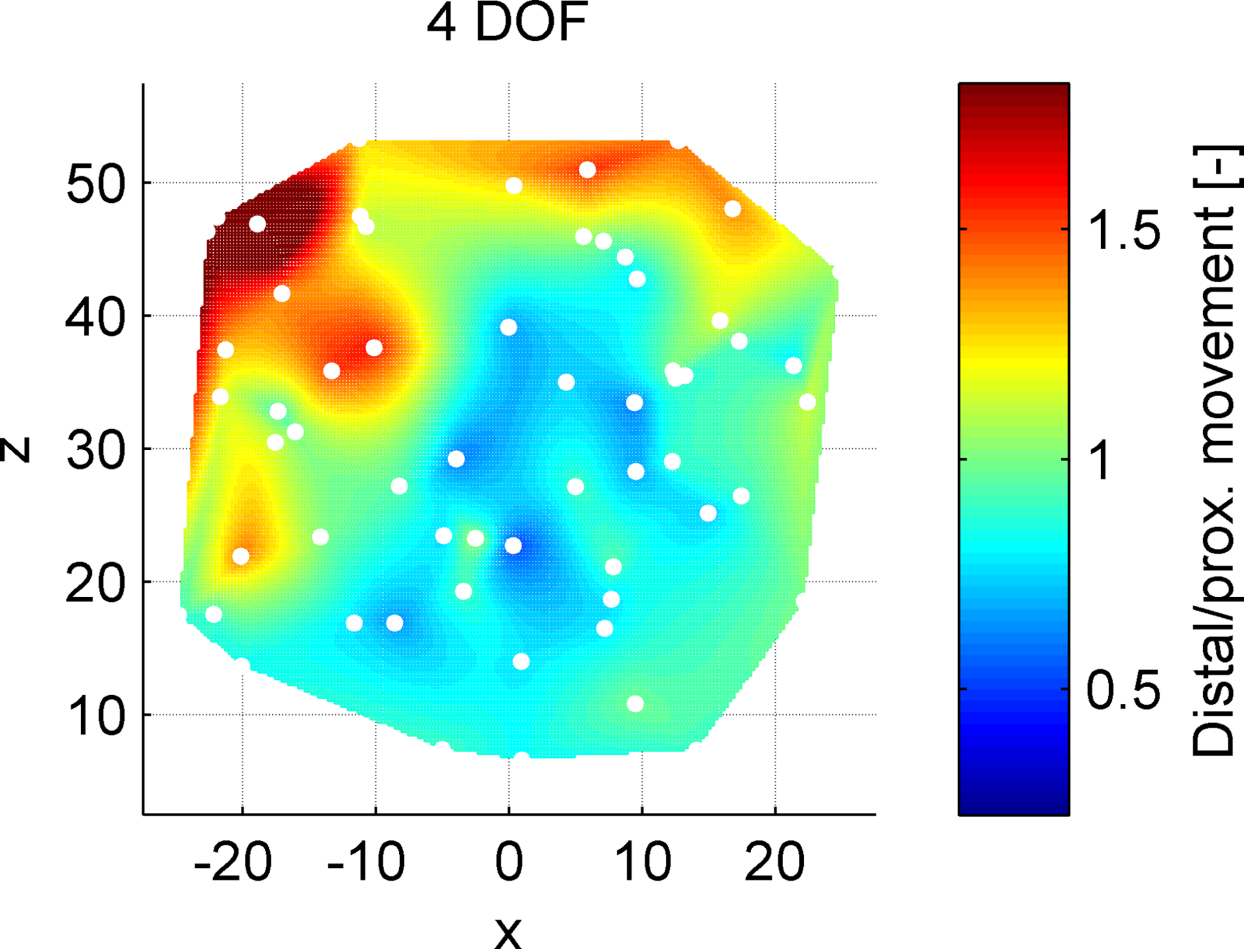
Number of distal segment movements divided by the number of proximal segment movement for the 4DOF control strategy. Data were normalized to the learning curve and target position within the workspace. The white dots represent the locations of the targets throughout the workspace.

Fig 8 shows the number of distal segment movements divided by the number of proximal segment movements for the 4DOF control strategy. These data were normalized as described above. The areas corresponding to a value smaller than 1 represent targets that were reached with more proximal than distal segment actuation, and areas with values greater than 1 represent the reverse. For the 2DOF control strategy, this value is by definition 1 because the frontal and distal segments have equal joint angles; hence in Fig 8, only the 4DOF control strategy is shown.

The left targets and the top targets were associated with distal segment actuation (Fig 8). The left targets require full index finger flexion, whereas the top targets require full index finger abduction. The other edges of the workspace lie closer to the resting position of the index finger for the hand posture. The blue area thus shows that in the largest part of the workspace the thumb was used for control; the index finger was used solely when required for approaching distant targets.

### Simultaneous segments actuation

Evaluation of simultaneous segments actuation, that is, when both the proximal and distal segments are actively used at the same time, indicates whether participants employed a stepwise or integrated way of controlling the segments. For 2DOF control, thumb movements are identically mapped to both the proximal and distal segment, all movements of both segments thus being simultaneous by definition. For 4DOF control, simultaneous segments actuation entails the concomitant use of the thumb and index fingers. For the 2DOF and 4DOF control strategies, simultaneous segments actuation was detected for 74.4% (SD = 7.9%) and 30.2% (SD = 7.9%) of the task durations, respectively. This difference is statistically significant (*t*(13) = 25.25, *p* < .001). Note that not 100% of the time simultaneous segments actuation were detected for the 2DOF control strategy, because of downtime in the movement of the thumb (i.e., 74.4% of the time the thumb was moving, and the remaining 25.6% of the time, the thumb was passive). For the 4DOF control, 30.2% of the time the thumb and index fingers were used simultaneously, and the remaining 69.8% of the time is spent moving only one or neither of the segments. The observed 30.2% simultaneous segments actuation at 4DOF control indicates a predominantly stepwise control method of participants, where (based on participant feedback as well as the recorded data) participants first used the thumb for gross instrument movement, and whenever possible also for fine-tuning control. The index finger was solely used when required. Fine-tuning control with the index finger is not a preferred control method for most of the participants, even though some participants indicated that they did try to use this strategy in later rounds.

The simultaneous segments actuation as a percentage of the task duration achieved by the first author after extensive training for the 2DOF and 4DOF control strategies were 78.0% and 55.1% respectively. Comparing this to the participants’ results shows for the 2DOF control an approximately similar instrument downtime (i.e., 25.6% participant downtime vs. 22% downtime of the first author). For 4DOF control, however, 24.9% more simultaneous segments actuation was observed (i.e., 30.2% participant vs. 55.1% first author simultaneous segments actuation). This higher degree of simultaneous segments actuation is likely to underlie the faster task completion times of the first author as compared to those achieved by the participants who had no more than 5 rounds of training (see Fig 4 and Fig 5).

No significant influences of round number, learning, or target location within the workspace were observed for the measured participants’ simultaneous segments actuation at 4DOF control. The fact that round number shows no substantial correlation with simultaneous segments actuation suggests that this strategy requires extensive training before adoption. Finally, an influence of distance to next target was present in the participant data, with 24.2% (SD = 17.5%) simultaneous segments actuation for the 10 mm distance, and 29.4% (SD = 16.9%), 32.8% (SD = 17.2%) and 33.3% (SD = 15.8%) for the 20 mm, 30 mm, and 40 mm distances, respectively. Thus, with increasing target distance, slightly more simultaneous segments actuation is observed. These results could be because nearby targets can be reached with only a single joint segment, negating the need for simultaneous segment actuation. A similar effect was observed for the first author’s performance data.

### Workload

**Fig 9:**
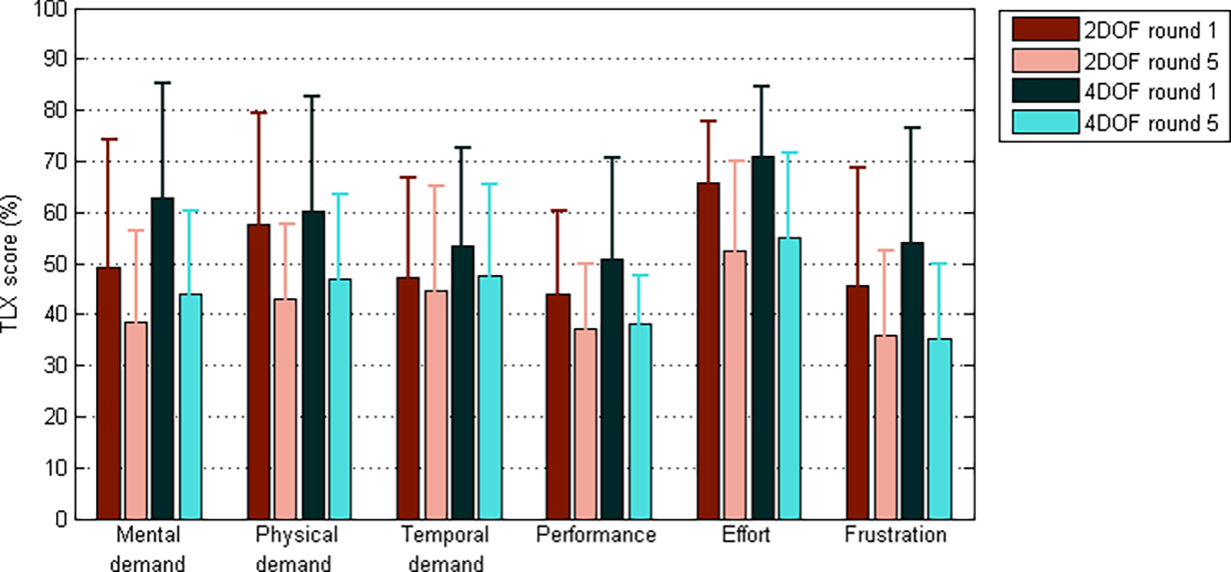
Raw NASA Task Load Index (TLX) item scores for every round and both control strategies.

**Fig 10:**
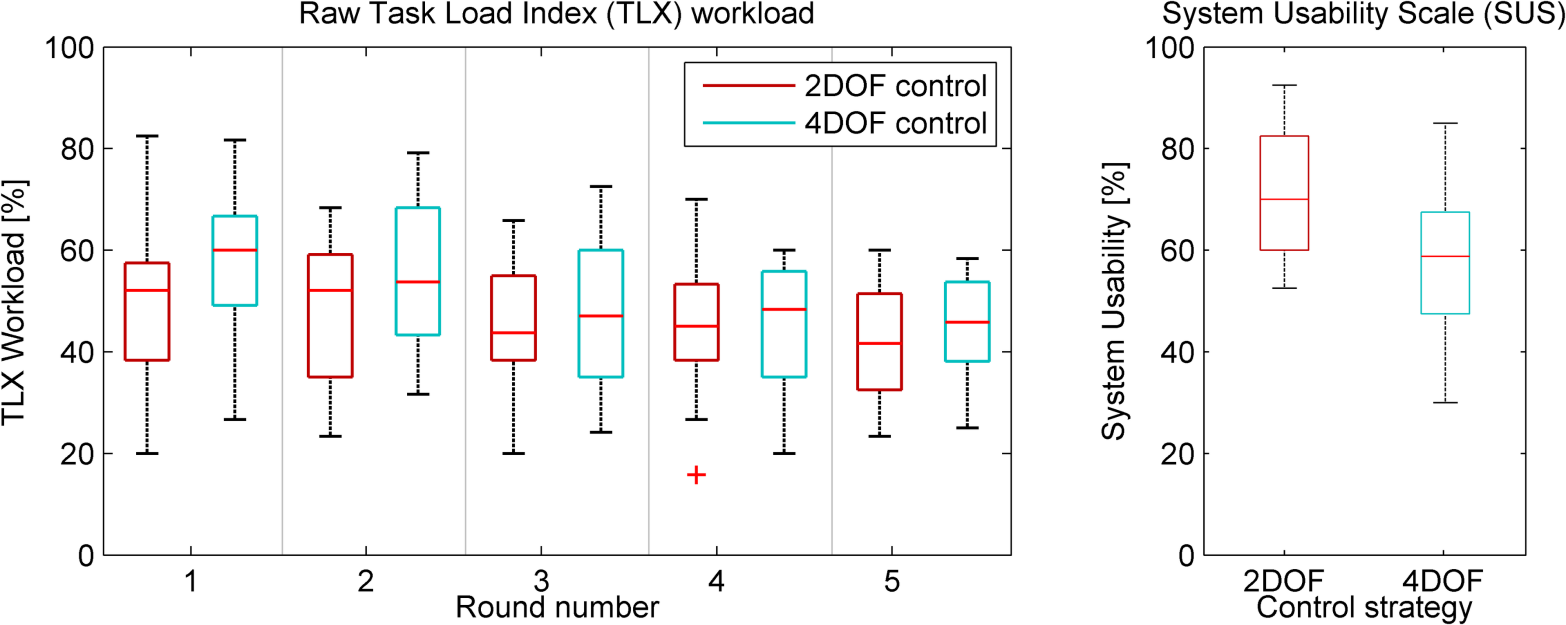
**Left: NASA TLX scores as a function of round number and control strategy.** Right: System Usability Scale (SUS) results for both control strategies.

NASA Task Load Index (TLX) assessment on the items (Mental Demand, Physical Demand, Temporal Demand, Performance, Effort, Frustration), overall workload as a function of round number, and the System Usability Scale (SUS) are provided in Fig 9 and Fig 10. The TLX workload decreases with round number, where the 4DOF control strategy shows a higher initial workload, but decreases to a similar level as observed for the 2DOF control strategy. No significant interaction was observed between control strategy and round number on TLX score (*F*(4,48) = 1.49, *p* = .220).The difference between control strategies are, however, statistically significant for the first two rounds (round 1: *F*(1,12) = 8.10, *p* = .015, round 2: *F*(1,12) = 7.42, *p* = .018). Moreover, at the 4DOF control strategy, the TLX scores for the first two rounds are statistically significantly higher than the subsequent rounds. The SUS scores for the 2DOF and 4DOF control strategies were 71.6% (SD = 13.0%) and 58.2% (SD = 14.9%), the difference being statistically significant (*t*(13) = 2.61, *p* = .021).

## Discussion

The goal of the presented research was to introduce and provide a proof-of-principle evaluation of, a new gesture-based design tool to evaluate multi-DOF control strategies. The hand tracking measurement setup, coupling hand- and finger motions to a simulated instrument, encompasses this design tool, enabling, for example, the comparison between the 2DOF and 4DOF control strategies. To discuss the value of the presented design tool, we first take a look at the design implications for the evaluated control strategies, and what we may learn from the results.

### Design implications

Based on the results gathered with the new design tool, the proposed design for 4DOF instrument control can be evaluated. A prominent question is whether humans can control the 4DOF instrument at a performance level similar to that of the 2DOF instrument. Considering that this study is only simulating positioning tasks, the results need to be interpreted with caution. Nevertheless, it can be seen that although the 4DOF control was initially challenging, there was a strong learning effect, and participants eventually reached a task performance that was only slightly slower than for the 2DOF control. The observed performance of the first author after extensive training suggests the two control strategies allow for equal performance on the current positioning tasks.

It is possible to make adjustments to both the input and output measures. To reach top-right targets in the workspace for example (see Fig 6), the magnification gain from input to output could be increased. Such an increase would allow for a smaller required thumb flexion and index finger abduction to reach the targets, potentially at the expense of reduced working speed and hit rate [57].

Considering the observed simultaneous segment actuation, it is apparent that participants require extensive training to master using both the thumb and index finger at the same time. The measured performance of the trained first author of this study, however, indicates that the simultaneous segment actuation strategy may be viable. In the current task, only the position of the instrument-tip was of importance in touching the target sphere. The next step in evaluating the 4DOF controlled instrument should be to simulate combined position-orientation tasks, to judge participants’ performance in orienting the instrument tip. Additionally, considering the laparoscopic camera was kept static during the current tasks, a higher fidelity trial in which this camera is actively controlled may provide additional insights (e.g., increased workload) into the 4DOF control strategy. This camera may even be controlled by a second person, as is generally the case in laparoscopic and endoscopic surgery.

### Alternative control strategies or instrument designs

Different levels of performance were observed in different planes. These performance differences may be because the magnification gains from fingers to instrument motions were not equal between the axes and fingers, as the fingers do not have equal ranges of motion. Observing the index finger at the 4DOF control (Fig 7, right), it can be seen that the targets in the top positions were the most challenging to reach. This is sensible in light of the rather small abduction range of the index finger that people have in general. The relatively large magnification gain (2.8) from index finger ab-adduction input (20 deg range) to vertical instrument segment deflection (56 deg range), leads to significant strain on the index finger to control it accurately, which raises the question whether this coupling strategy of linking the index finger ab-adductions to vertical instrument motions should be used at all. Perhaps it is preferred to use only index finger flexion for horizontal distal instrument segment control, and to couple vertical distal segment control to thumb flexion. In essence, this amounts to 3DOF control, with thumb flexion coupled to overall vertical instrument bending, and thumb ab/adduction and index finger for segmented horizontal bending.

More substantial design alterations may also be investigated in future research. For example, it is possible to switch the control couplings, so that the index finger controls the proximal segment, and the thumb the distal segment. Considering the preference of participants in using the thumb, another control option is to use this finger for both segments but to allow for discrete switching between simultaneous and single-segment actuation. One embodiment may be to use index finger flexion as a discrete control switch (like pressing a button) to alternate between simultaneous segment control (i.e., the 2DOF control strategy used in this study) and distal segment control (while locking the proximal segment in place). This two-step approach is likely to yield relatively slow task performance but may also yield improved positioning accuracy.

### Design tool limitations and considerations

By implementing the design tool presented in this study, far-reaching design alterations concerning instrument DOF and control strategies may be evaluated without the need for prototyping. The advantages of the measurement tool, however, comes at the cost of several limitations, the largest of which being the lack of tactile cues and haptic feedback otherwise present when handling a physical prototype. Not being able to feel the boundaries of the instrument’s workspace naturally, for example, as would otherwise be the case when handling joysticks, forces one to identify and continually keep track of their finger input motions regarding these boundaries. Haptic feedback, moreover, in laparoscopic instrumentation is an important field of study, considering that minimally invasive instrumentation masks force cues [58]. Haptic feedback is of particular use for feeling differences in tissue consistencies, applied pressures, and limiting strain in surgeon’s hands [59]. Considering the setup currently does not simulate the tactile sensation of holding and using the instrument, nor any of the task-related forces, design considerations to this end cannot be assessed. However, because the Nimble VR measurement system relies on the Kinect’s infrared depth camera, infrared transmitting objects (such as the custom Plexiglas support handle used in this study) may be implemented without significantly influencing hand postural estimates. As such, tactile cues and sensations may be approximated using props, though likely at a low level of fidelity.

The 500 ms measurement latency of the system in measuring finger ab/adduction affected participants to mostly adopt an effective “move-and-wait” strategy to cope with the delay. This delay influences the results to an extent, so that measurement results deviate from those that would be obtained with real prototypes. To exemplify, one may compare our observed mean task time of 3.34 s (last round of 2DOF control task, see Fig 4 left) to the average 0.98 s task time in the study of Fan et al. [60]. Fan et al. used a physical, 2DOF thumb-controlled, instrument (Microflex, DEAM, Amsterdam, NL [61]) for positioning tasks that required shaft and steerable tip control. Although the tasks are not identical, the main task aspect is the control of the 2DOF tip, which shows to be substantially faster in practice than in our measurement tool. Accordingly, on account of the latency issue, the tool is best suited to compare control strategies relative to each other and to assess to which extents participants can cope with complex controls. More research should be devoted to understanding the main performance differences between a physically prototyped instrument and our virtual equivalent.

The aim of the introduced design tool in this study is to expedite control developments for multi-branched and multi-steerable instrumentation. It is not meant to replace a full prototype control evaluation. The design tool is best suited to aid in the preliminary evaluation of envisioned but untested control methods and settings. However, the quality of the hand tracking used in this setup may still be greatly improved. One way to solve the issue of the measurement latency on the finger ab/adduction measurements is to upgrade the used 5DT Data Glove to a 5DT Data Glove 14 Ultra, which incorporates sensors between the fingers for ab/adduction measurements [51]. However, their placement may be too obtrusive for natural finger motions. Considering the current rapid advances made in consumer electronics for hand motion tracking (e.g., Leap motion sensor [62, 63]), it is likely that technological developments towards Virtual and Augmented Reality [64, 65] will benefit the control evaluation design tool as presented in this study.

## Conclusions

A design tool was presented to evaluate multi-DOF control strategies for minimally invasive medical instrumentation. A proof-of-principle evaluation was performed, comparing a 2DOF steerable tip, controlled with the thumb, to a 4DOF steerable tip (two serially stacked 2DOF segments), controlled with the thumb and index finger. Results show that the design tool provides the ability to evaluate instrument control performance regarding task time and learning effects, as well as differentiate performance metrics to travel distances between targets and their respective locations within the instrument workspace.

The proof-of-principle evaluation, based on simulated positioning tasks, showed the 4DOF control strategy to have a stronger learning effect but to eventually perform only slightly slower as compared to the 2DOF control strategy. Results further indicate that participants favor the use of the thumb in both gross and fine-tuning movements. Additionally, index finger ab/adduction as input motion to control the instrument tip is found least functional and may be negated in future instrument design. Simultaneous segment actuation at the 4DOF control strategy, i.e., using both the thumb and index fingers simultaneously, is proven to be challenging for participants, but also a viable control strategy.

Based on the results, and without having to resort to prototyping, the new gesture-based design tool has proven effective in identifying possible improvements for the assessed control strategies as well as identifying potential new control strategies.

## Supporting information

S1 Video Gesture-based instrument design tool implementation in the performance of positioning tasks with the 2DOF and 4DOF control strategies. Two video feeds, showing the execution of a 100 consecutive tasks with both control strategies, are shown side by side (left 2DOF, right 4DOF). The target sequences at both control strategies are identical. A video feed is paused once a target is reached and resumed once both control strategies have successfully reached the target. A running clock on both video feeds showcases the cumulative time difference between control strategies as the sequence of tasks is performed. The video shows the performance of the first author of this publication. Data corresponding to the shown task performance was not collected.
(AVI)
